# Sand Flies Control: A Review of the Knowledge of Health Professionals and the Local Community, Province of El Hajeb, Morocco

**DOI:** 10.3390/ijerph17228448

**Published:** 2020-11-15

**Authors:** Karima El-Mouhdi, Abdelkader Chahlaoui, Samia Boussaa, Mohammed Fekhaoui

**Affiliations:** 1 Geobiodiversity and Natural Patrimony (GEOPAC), Scientific Institute, Mohammed V University, Rabat, Morocco; karima_elmouhdi@um5.ac.ma (K.E.-M.); medfekhaoui@gmail.com (M.F.); 2 Natural Resources Management and Development Team, Laboratory of Health and Environment, Faculty of Sciences, Moulay Ismail University, Meknes, Morocco; a_chahlaoui@yahoo.fr; 3 ISPITS-Higher Institute of Nursing and Technical Health Occupations, Ministry of Health, Marrakesh, Morocco

**Keywords:** sand flies, health risk, vector-borne diseases, knowledge assessment, entomoprophylaxis, Morocco

## Abstract

Sand flies are insect vectors of several diseases including leishmaniases. These vector-borne diseases represent a public health problem in several countries around the world, including Morocco. The objective of this study was to assess simultaneously the knowledge of health professionals and inhabitants on sand flies; a cross-sectional survey was conducted between April and June 2019 among 424 people, 34% of whom were health professionals and 66% of whom were inhabitants of the province of El Hajeb in central Morocco; 46.3% of doctors, 50.7% of nurses, 66.7% of midwives and 69.4% of inhabitants showed a low knowledge of sand flies. Most participants believed that sand flies breed in stagnant and polluted waters. Negative attitudes were found among 72.2% of the inhabitants. Factors associated with a high level of knowledge included continuing education among health professionals and information on vector-borne diseases among residents. The conceptual and cognitive gaps in the knowledge of sand flies reflect the lack of information and training on sand flies. The results of the sand fly knowledge review can be integrated into the national leishmaniases control program and the integrated vector management strategy to raise public awareness on the health risks of sand flies.

## 1. Introduction

Sand flies (Diptera: Psychodidae), are small biting insects that transmit parasitic (leishmaniasis), bacterial (bartonellosis) and viral (rift valley disease virus) diseases. Leishmaniases as a group of vector-borne diseases alone account for 17% of infectious diseases, with more than one million deaths each year [1]. In Morocco, these insects are abundant and widespread throughout the country [2]. They are responsible for the transmission of serious and deadly parasitic diseases such as visceral leishmaniasis and other disfiguring diseases such as cutaneous leishmaniasis [3,4]. These parasitic diseases represent a major public health problem in Morocco [5]. They are endemic in different regions of this country [6,7,8]. In fact, the North has historically been known as an epidemic focus of visceral leishmaniasis with *Leishmaniainfantum* (*L. infantum*) and the South has been recognized as an active focus of cutaneous leishmaniasis with *Leishmania major*, while the center of the country has been characterized by the cohabitation of both cutaneous and visceral forms [6,9,10]. The spread of these vector-borne diseases is closely linked to the climate and the geographical expansion of the sand flies vector species [8,11,12].

In 2016, the World Health Organization (WHO) classified Morocco as an endemic country with a high incidence of cutaneous leishmaniasis with the population at risk of 14% [13]. The epidemiological situation of these diseases has been of growing concern since 1997 when the national program to combat leishmaniasis was officially launched to address the spread of the disease in Morocco.

According to the Ministry of Health, the number of new cases of leishmaniasis is increasing with a predominance of cutaneous leishmaniasis due to *L. major* (54%) of which rodents are the reservoirs, followed by cutaneous leishmaniasis due to *L. tropica* (43%) of which men are the reservoirs, and finally visceral leishmaniasis due to *L. infantum*, of which dogs are the main reservoir [14]. The identification of the *Leishmania* species involved has allowed the description of the geographical distribution of the forms of leishmaniasis on the whole Moroccan territory. Consequently, the species most identified in the North is *L. infantum*; in the center, it is *L. tropica*, while in the South it is mainly *L. major* [3]. At present, the health authorities state that the rate of cases detected at the level of health facilities do not exceed 35% of the total estimated cases at the national level, despite the efforts made within the framework of the national leishmaniases control program [14].

Indeed, sand flies as vectors of human and animal leishmaniasis are small social midges that survive near human dwellings and animal shelters, where they feed on the blood of dogs and other vertebrate mammals. Nevertheless, studies have revealed that leishmaniases and their vectors are still the most neglected and least known among people [15,16,17]. In this framework, *Phlebotomussergenti* was proved responsible for the transmission of leishmaniasis to *L. tropica*, and *Phlebotomuspapatasi* was identified as the vector of cutaneous leishmaniasis to *L. major*, while the species *P. perniciosus, P. ariasi* and *P. longicuspis* was incriminated in the cycle of transmission of cutaneous and visceral leishmaniasis to *L. infantum* [8,17,18]. Thus, given the absence of vaccine prophylaxis, the complexity of the chain of transmission, the diversity of the vector and the multiplicity of the parasite, the disease continues to spread to other regions and individual protection against the vector remains the best means of preventing leishmaniasis [19].

From an epidemiological and public health point of view, it is important to improve the level of knowledge and attitudes of individuals towards the disease vector to ensure the success of a prevention and control program. In this sense, the purpose of this document is to assess the local community’s knowledge and attitudes towards sand flies and to examine the knowledge of the health professional as a key factor in the implementation of the prevention program activities. The results of this study will enable decision-makers to adapt control activities to the socio-cultural context of the affected population.

## 2. Materials and Methods

### 2.1. Study Area

A cross-sectional survey was conducted among health professionals and the local community in the province of El Hajeb in central Morocco during April and June 2019. El Hajeb (33°41′ N, 5°22′ W) has 16 communes (12 rural and 4 urban) and its population was estimated at 247,016 inhabitants in 2014 [20]; with the altitude of 1000 m and a temperate climate [21].

Epidemiologically, El Hajeb province borders the provinces of Sefrou and MoulayYacoub, endemic foci of cutaneous leishmaniasis [6,7,22]. Studies have revealed the coexistence of both forms of leishmaniases in El Hajeb Province, with cutaneous leishmaniasis (81%) predominating over visceral leishmaniasis (19%). The first age of the disease infestation, in autochthonous cases, was 24 months for the cutaneous form and 13 months for the visceral form [9]. A recent study revealed that 75% of the leishmaniases cases reported at the provincial level are indigenous cases from rural areas, 62% of which were reported in the rural communes of Bitit and Laqsir, which border the province of Sefrou [9].

In terms of health, the health care offered in ELHajeb is mainly public, with the private sector, limited to a few pharmacies and informal providers [20,21,23]. The public health establishments consist of a hospital network (a provincial hospital and a hemodialysis center), and a primary health care network with 23 health centers (12 are rural health centers, 6 are rural dispensaries, 5 are urban health centers). In the year 2019, the number of health personnel working in the province’s public health structures was 248, divided between health professionals (63 medical, 185 paramedical) and administrative staff (45) [23].

### 2.2. Study Design and Sampling

This study was conducted in two stages to collect the required data. The first was to select communities affected by the disease to increase the chances of obtaining responses from people who had seen or had a history of skin lesions due to leishmaniasis and the second was to determine the sample for the study. The selection of localities was based on their history of exposure to cutaneous or visceral leishmaniasis. These data were taken from the annual surveillance reports of the cases reported to the health authorities of the El Hajeb health delegation and from the study of the epidemiological situation of leishmaniases during the years 2013–2017 in the province [9]. All cases of cutaneous and visceral leishmaniases were reported in the following municipalities: Bouderbala, Agourai, Bitit, Iqdar, Laqsir, ELHajeb, SabaaAyoune and AinTaoujdate. These communes were selected to carry out the exploratory survey on the popular knowledge of sand flies in the province of El Hajeb in central Morocco.

The inclusion criteria for the local population were mainly their willingness to participate in the study; to be an adult, a resident and a native of ELHajeb. To have a representative sample, a door-to-door convenience sampling method was adopted to collect data from the inhabitants of the selected localities and only one respondent per household was selected.

For health professionals, all doctors, nurses, midwives and health technicians working in the region’s health structures (23 health centers and two hospitals) who participate in care activities were included in the study. Convenience sampling was used to recruit health professionals who wished to participate in the study [24].

### 2.3. Data Collection

The data were collected from April to June 2019 from the inhabitants of the selected localities and all health personnel working in the public health structures. The two tools that were used to collect the data were a self-administered questionnaire for health professionals and an assisted interview (in Arabic) for the inhabitants. The design and construction of these tools were carried out following the guidelines of Khan et al. [25] and Frary [26].

For both tools, there were four sections: the first one to collect socio-demographic variables, the second one to collect experience with the disease (as a caregiver in the questionnaire or as a patient in the interview), the third to assess the knowledge of the participants about the biology and ecology of sand flies and the fourth to examine the measures used for prevention. Our data collection tools were validated by a pre-test with 21 inhabitants for the interview and 15 health professionals for the questionnaire who were not part of the selected sample.

### 2.4. Data Processing and Analysis

The data were entered into Excel 2007; validation of the datasets was performed to detect inconsistencies, and then transferred to SPSS v23 for further analysis. Descriptive and logistic regression statistics were performed. Associations between variables were assessed using Chi-Square tests. *p* < 0.005 was considered statistically significant.

The level of knowledge was assessed based on the participants’ responses. Items were coded with (1) for “correct” responses and (0) for “incorrect” responses. Scores were calculated for each respondent for the different items of the data collection tools and then a composite score (out of a total of 4 points) was assigned. These scores were analyzed descriptively. For the population, the scores ≥3 meant that knowledge was sufficient, while scores ≤2 meant that knowledge was insufficient. For health professionals, scores ≥4 meant that knowledge was high, while scores <2 meant that knowledge was low. Thus, a score between 2 and 3 meant that the levels of knowledge about sand flies were medium.

### 2.5. Ethical Considerations

The study was authorized by the provincial delegation of the Ministry of Health of the Province of ELHajeb. All participants were informed about the objectives of the study and the confidentiality of their identities. They were free to accept or refuse to participate in the study [27]. After each interview, all participants were given information about the sand flies and the diseases that can transmit, their breeding places and biting times, and recommended ways to avoid their risks.

## 3. Results

### 3.1. Health Professionals

#### 3.1.1. Socio-Demographic Characteristics and Levels of Health Professional Knowledge of Sand Flies

The socio-demographic characteristics of health professionals are presented in Table 1. Of the 248 health professionals working in public health structures in ELHajeb province, 143 completed the questionnaire. The participation rate was 58%. More than half (53.1%) were women. The age group of 31–40 years was the most dominant (47.6%).

In terms of professional categories, nurses made up more than half of the study population (51%), followed by physicians who accounted for 28.7%, then midwives (14.7%) and health technicians (5.6%). In addition, 63% of these staff worked in primary health care facilities, while the remainder worked at the provincial hospital level (37.1%). By area, 46.9% in rural areas compared to 53.2% in urban areas.

In terms of years of work experience, 37.8% of the participants had between 10 and 15 years of seniority, while 11.9% had more than 20 years of work experience in the health service and only 4.2% had less than 5 years of seniority. Follow-up of continuing education on vector-borne diseases was confirmed by 16.8%.

Regarding the overall knowledge of sand flies, more than half of the participants showed a low level of knowledge (51.7%). By professional category, the highest levels of knowledge were recorded among doctors (14.6%), followed by health technicians (12.5%), then nurses (9.6%) and finally midwives (4.8%).

The knowledge of the sand flies as a true vector of leishmaniases was achieved by 51% of the health professionals, while 49% thought that *Anopheles* are the vectors of the disease (Table 2). Only 36.4% of the respondents had correctly identified the sand flies breeding sites, compared to 63.6% who thought that sand flies breed in stagnant and polluted waters. Regarding the biting time, few respondents (19.6%) linked it to the activity of sand flies, which is higher between dusk and dawn. Thus, more than half (55.9%) of the participants stated that the best methods to protect themselves against the risk of sand flies is the use of insecticide-treated mosquito nets.

#### 3.1.2. Factors Associated with the High Level of Health Professional Knowledge of Sand Flies

The results of the logistic regression analysis are presented in Table 3. They show that several factors were found to be significantly associated with low knowledge. Indeed, the bivariate analysis revealed that low levels of sand flies knowledge were strongly associated with the absence of continuing education (*p* = 0.001), professional seniority of fewer than 5 years in the health services (*p* = 0.009) and belonging to an age group between 20 and 30 years (*p* = 0.007).

Multivariate analysis revealed that male physicians (*p* =0.003) who treated patients with cutaneous leishmaniasis lesions (*p* = 0.007) and who had undergone continuing education on vector-borne diseases (*p* = 0.003) were more likely to show higher levels of sand flies knowledge. Female nurses (*p* = 0.007) who had provided care to persons with cutaneous leishmaniasis (*p* = 0.002) and worked in rural health facilities (*p* < 0.001) and had received continuing education on vector-borne diseases (*p* = 0.010) showed high levels of knowledge.

For midwives, regression analysis revealed that those who were over 40 years of age (*p* =0.035) and had more than 20 years of experience in health services (*p* = 0.024) were the least likely to show low knowledge of sand flies.

### 3.2. The Local Community

#### 3.2.1. Socio-Demographic Characteristics of the Local Community

The socio-demographic characteristics of the local population were presented in Table 4. A total of 281 people were interviewed, ranging in age from 17 to 67 years, with an average age of 38.84 years. Overall, 55.2% of the participants lived in rural areas and 68% of the respondents were women. The illiteracy rate was 31.3% and more than half of the participants were people with low socio-economic status who were beneficiaries of a social security scheme for the economically disadvantaged (RAMED).

Concerning information on insect-borne diseases, 35.2% of the respondents were informed, of which 11% were informed by friends/neighbors and 10.3% by means of television.

#### 3.2.2. Factors Associated with Sufficient Knowledge and Positive Attitudes of the Community Regarding Sand Flies

Factors associated with sufficient knowledge and positive community attitudes towards sand flies are presented in Table 5. Of the 281 respondents, 63.3% (178) reported having seen the lesion before when shown a picture of leishmaniasis skin lesions. However, 59.6% of the respondents stated that this type of lesion was known as “Hbob” from “Chniwla” or “Hbob” from “Namos”.The term “Hbob” in Moroccan dialect means pimples on the skin. While the words “Chniwla” and “Namos” refer to biting flying insects. Specific questions about the vector were asked (Table 2). Indeed, the level of overall knowledge about the sand flies was insufficient in 69.4% of the respondents. For example, only 12.8% knew that sand flies activity is increased at sunset and dawn. Few of the respondents (29.9%) stated that the favorable environments for the proliferation and multiplication of sand flies were disposal sites for waste and manure. While 70.1% of the participants thought that sand flies reproduce from stagnant and polluted waters.

The results of the logistic regression (Table 5) showed that several factors were significantly associated with sufficient overall knowledge. Indeed, the analysis revealed that the females (*p* = 0.021) who lived in a rural area (*p* < 0.001), had a history of cutaneous leishmaniasis (*p* < 0.001) and had at least secondary education (*p* = 0.030) were associated with sufficient knowledge.

Attitudes were negative in 70.1% of the participants; 27.8% declared that they used methods to protect themselves against insect bites such as plants, especially basil (5%), insecticide bombs (7%) and mosquito nets (3%). While 72.2% said they did not adopt any means to protect themselves. In the case of stings and the appearance of skin lesions, 45.7% preferred to apply traditional remedies while others would consult a healer (17.5%). Only 22% said they seek treatment from a health professional or 14.8% from a pharmacist. Higher education (*p* < 0.001) and having received information on insect-borne diseases (*p* = 0.003) were the only factors significantly associated with a positive attitude. Thus, sufficient knowledge was significantly associated with positive attitudes (*p* = 0.031).

## 4. Discussion

Authors should discuss the results and how they can be interpreted in the perspective of previous studies and of the working hypotheses. The findings and their implications should be discussed in the broadest context possible. Future research directions may also be highlighted.

Sand flies are small hematophagous insects that are very common throughout the world. The health risks of these insects are linked to their vectorial capacity to transmit several diseases. In Morocco, sand flies are responsible for the transmission of cutaneous and visceral leishmaniasis, which are endemic in different regions of the country. This study was conducted in the province of ELHajeb in central Morocco. The ultimate goal was to examine people’s knowledge of sand flies to help officials plan awareness and prevention programs according to the context of our country.

To our knowledge, this study was the first in Morocco to report the simultaneous evaluation of the knowledge of the local community and health professionals about the sand flies as a vector of leishmaniases in our country.

Despite its subjectivity, the methodology used in this work remains the most suitable to study the perceptions and attitudes of a population [28]. However, the high level of participation of the inhabitants and professionals is considered among the strong points of the study.

In addition, the survey of health professionals has made it possible to integrate the main categories of health professionals (doctors, nurses, midwives and health technicians) and all health care institutions in the province (hospitals, rural dispensaries, urban and rural health centers). On the other hand, women represented 53.1% among health professionals and 68% among the local community respondents in our study. The womenwere more numerous than men in health care institutions according to the latest statistics from the Ministry of Health [23]. They are also the most likely to be available in their homes according to Moroccan traditions [20] and are the most influential in the family in matters of health [29]. Indeed, according to the latest general population census of 2014, women represented more than half (50.5%) of the Moroccan population [20].

All of these elements are in favor of the possibility of generalizing our results to other regions of the country or even to other areas where the socio-demographic and professional characteristics are similar.

Comparison and analysis of the results obtained from the local population and health professionals have enabled us to identify points of divergence and convergence in their knowledge of sand flies. The results of the knowledge review revealed low and insufficient knowledge of the sand flies among health professionals and the local population. Indeed, several conceptual and cognitive gaps were revealed in the knowledge of the vectorial capacity of the sand flies, its breeding places and biting times, negative attitudes in the application of preventive measures and ways to protect oneself against the risks of sand flies.

Every day, studies are carried out on leishmaniases around the world. The epidemic and biological aspects of the disease have been sufficiently described. The latest WHO recommendations encourage researchers and scientific communities to pay more attention to the socio-cultural aspects of the disease, in particular, the relationship between humans and insect vectors, to increase people’s awareness of entomoprophylaxis and to correct risk behaviors [4]. Given that risk increases with poor adherence to preventive measures [4]. It is therefore important to determine how local communities perceive and understand vector-borne diseases, as this can have a direct impact on disease behavior.

The first finding revealed by this study is that the sand flies were recognized in the local population of El Hajeb in central Morocco under the names “Chniwla” and “Namos”. However, this popular name was only recognized by a few health professionals. These popular terms were also encountered by Bennis and his colleagues [30] in the provinces of Er-Rachidia and Tinghir in southern Morocco. This said, there is no difference in the popular terminology used to refer tothe sand flies in central and southern Morocco. On the other hand, our study suggested to take into consideration these popular concepts and to integrate the Moroccan socio-cultural context for the effectiverealization and concretization of the citizens’ awareness of the risks. Indeed, the integration and use of terminology in health education and prevention programs will make it easier to establish channels of communication between the population and health professionals, to make the sand flies known to a large number of the public and to ensure community participation in vector control activities.

An interesting observation was also raised concerning the lack of knowledge of the sand flies as the only vector responsible for the transmission of leishmaniases in Morocco. Indeed, more than half of the participants had a false impression that mosquitoes caused leishmaniases. This could be explained by the importance given to *Anopheles* mosquito control activities in favor of the sand flies. Additionally, *Anopheles* mosquito control under the malaria control program was largely successful throughout the country, and since the elimination of malaria in 2012, surveillance of larvae has been intensified [29]. This finding is similar to that of a study conducted by Ruoti and colleagues [30]. In Paraguay among the local community and health professionals, they found that the majority of doctors, nurses and care assistants believed that leishmaniases is also transmitted through the bite of infected mosquitoes due to the intensification of dengue control activities.

Furthermore, our results revealed that 70.1% of the local population believed that sand flies reproduce in aquatic environments. This corroborates with Akram’s study [31] carried out among the local community of Punjab in Pakistan.

Negative attitudes (70.1%) were observed in the local community of El Hajeb. Most (72.2%) said that they did not adopt any means to protect themselves. The rest used plants such as basil (5%), insecticide bombs (7%) and mosquito nets (3%) to avoid insect bites. However, WHO confirms that long-lasting insecticide-treated nets are the most effective in preventing leishmaniases and avoiding the risk of sand flies bites [27]. In addition, entomologists have argued that appropriate protective measures against sand flies include environmental hygiene, permanent cleaning of animal habitats, proper disposal of household and organic waste and removal of domestic animal habitats outside the home, which are essential to prevent the risk of sand flies coming into contact with humans [32]. Other specialists in the field have argued that the application of traditional methods in preventing the risk of sand flies bites, such as the use of plants instead of mosquito nets, could be detrimental to the adaptation of preventive measures related to the correct management of the environment, the main interventions of which are aimed at better management of solid sanitation, disposal of waste and manure as breeding sites for sand flies larvae [33].

In fact, regression analysis revealed that good knowledge of sand flies is associated with several factors. Among the local community, sufficient knowledge and positive attitudes were associated with the educational levels of individuals and the opportunity to be informed. Indeed, the level of education of the individual is significantly positively correlated with the level of knowledge and positiveattitudes.

Positive attitudes towards pest control were found to be related to sufficient knowledge. Similarly, among health professionals, continuing education appears to play a very important role in improving knowledge, and is strongly related to high levels of knowledge for all professional categories. Seniority in the care service and experience in caring for people with leishmaniasis seem to be factors dependent on knowledge of the sand flies.

However, the majority of professionals have not received specific training. Because of their direct contact with the population, health professionals are in the best position to make citizens aware of the risks. Considering the results obtained, our study will suggest that it is necessary to implement continuous training and retraining sessions for health professionals so that they can properly inform the population at risk and encourage them to apply prevention and protection measures against the sand flies. The use of brochures and posters with pictures describing the sand flies, their health hazards and ways to avoid their risks is an effective strategy to shape knowledge and correct misinformation about sand flies. It is also suggested to use technology in awareness-raising and to integrate the concepts obtained regarding popular terminology to simplify scientific terminology, which will allow the messages to be easily assimilated by a large number of audiences.

From the analysis of all the results obtained, we were able to deduce that despite the almost permanent presence of the sand flies in Morocco, this phenomenon is not well known by Moroccans. The data collected reflect this lack of knowledge of the population and professionals about the sand flies and its characteristics concerning the mosquito, which most of the participants referred to as a knowledge base to describe the course of action to be taken in measures to control the sand flies. The use of stagnant water disposal as a control strategy was testimony to this confusion. This will negatively influence sand flies prevention and protection activities. The lack of continuous training, or even its absence and the concentration of control activities on the *anopheles* mosquito vector of malaria in the integrated vector control management activities are all factors that have contributed to this unsatisfactory state of knowledge about the sand flies vector of leishmaniases in Morocco.

## 5. Conclusions

This study revealed insufficient and weak knowledge of the sand flies as the only vector of leishmaniases. This lack of knowledge was mainly associated with the lack of continuous training for health professionals and the lack of information and awareness of the community on the health risks of this insect. The study potentially provides a baseline on the state of knowledge of sand flies, particularly in central Morocco. The results can be integrated into public awareness in at-risk areas and the conceptualization and implementation of a national health education strategy for the prevention of sand flies risks adapted to the socio-cultural context of the country

## Figures and Tables

**Table 1:** Socio-professional characteristics and levels of knowledge of health professionals about sand flies.

Variables		Total		Doctors		Nurses		Health Technicians		Midwives	
		*n* = 143	%100	*n* = 41	%28.7	*n* = 73	%51.0	*n* = 8	%5.6	*n* = 21	%14.7
Sex	Male	67	46.9	24	58.5	37	50.7	6	75	0	0
	Female	76	53.1	17	41.5	36	49.3	2	25	21	100
Age	(20–30)	14	9.8	4	9.8	7	9.6	0	0	3	14.3
	(31–40)	68	47.6	11	26.8	37	50.7	5	62.5	15	71.4
	(41–50)	41	28.7	19	46.3	16	21.9	3	37.5	3	14.3
	(+50)	20	14	7	46.3	13	21.9	0	37.5	0	14.3
	<5 years old	6	4.2	3	7.3	3	4.1	0	0	0	0
Seniority in the health service	(5–10)	41	28.7	8	39	17	41.1	3	37.5	13	23.8
	(10–15)	54	37.8	16	24.4	30	15.1	3	12.5	5	14.3
	(15–20)	25	17.5	10	24.4	11	15.1	1	12.5	3	14.3
	>20 years old	17	11.9	4	9.8	12	16.4	1	12.5	0	0
Working structure	Hospital	53	37.1	15	36.6	26	35.6	6	75	6	28.6
	Urban Health Centre	23	16.1	17	41.5	31	42.5	1	12.5	13	61.9
	Rural Health Centre	62	43.4	8	34.8	12	52.2	1	4.3	2	8.7
	rural dispensary	5	3.5	1	2.4	4	5.5	0	0	0	0
Continuing Training on Vector-Borne Diseases	No	119	83.2	32	78	59	80.8	7	87.5	21	100
	Yes	24	16.8	9	22	14	19.2	1	12.5	0	0
	High knowledge (score ≥ at 4)	15	9.8	6	14.6	7	9.6	1	12.5	1	4.8
Levels of general knowledge about sand flies	Average knowledge (score from 2 to 3)	51	35.7	16	39	26	35.6	3	37.5.	6	28.6
	Low knowledge (score < to 2)	74	51.7	19	46.3	37	50.7	4	50	14	66.7

**Table 2:** Knowledge on the biology and ecology of sand flies and means of control.

			Knowledge of the Sand Flies as a Vector of Cutaneous Leishmaniases		Knowledge of Sand Flies Breeding Grounds		Knowledge of Sand Flies Biting Times		Knowledge of Protection Methods		Total	
			*n*	%	*n*	%	*n*	%	*n*	%	*n*	%
Population (*n* = 281)		Yes	107	38.1	84	29.9	36	12.8	107	38.1	89	31.7
		No	174	61.9	197	70.1	245	87.2	174	61.9	192	68.3
Health Care Professionals	Doctors (*n* = 41)	Y	28	19.6	9	6.3	11	7.7	29	20.3	19	47.0
		No	13	9.1	32	22.4	30	21.0	12	8.4	22	53.0
	Nurses (*n* = 73)	Yes	36	25.2	30	21.0	14	9.8	40	28.0	30	41.0
		No	37	25.9	43	30.1	59	41.3	33	23.1	43	59.0
	Health technicians (*n* = 8)	Yes	3	2.1	3	2.1	2	1.4	4	2.8	3	38.0
		No	5	3.5	5	3.5	6	4.2	4	2.8	5	63.0
	Midwives (*n* = 21)	Yes	6	4.2	10	7.0	1	0.7	7	4.9	6	29.0
		No	15	10.5	11	7.7	20	14.0	14	9.8	15	71.0
	Total (*n* = 143)	Yes	73	51.0	52	36.4	28	19.6	80	55.9		
		No	70	49.0	91	63.6	115	80.4	63	44.1		

**Table 3:** Factors associated with high sand flies knowledge according to each occupational category.

Logistic Regression	Cœf.	OR ^1^	CI ^2^ à 95%	*p*-Value < 0.05
Doctors (*n* = 41)				
Age (41–50 years)	−0.54	2.006	0.18–1.87	0.291
Sex (Male)	−2.21	2.060	−1.54–1.94	0.003
Type of work structure (Rural Health Centre)	2.28	2.411	1.55–4.18	0.000
Seniority in the health service (15–20 years)	−3.5	2.297	−5.7–1.93	0.059
History of caring for people with cutaneous leishmaniasis	0.26	2.171	0.26–1.54	0.007
Continuing training on VBD ^3^	1.35	2.173	0.16–1.55	0.003
Knowledge of the sand flies	−2.51	2.202	1.38–2.11	0.006
Nurses (*n* = 73)				
Age (41–50 years)	0.99	1.104	0.67–1.8	0.000
Sex (Female)	2.78	1.072	1.70–4.69	0.007
Work structure (Rural Health Centre)	−1.15	1.190	0.74–1.91	0.000
Seniority in the health service (15–20 years)	0.67	1.069	−0.19–1.16	0.046
History of caring for people with cutaneous leishmaniasis	1.79	1.109	−0.32–0.60	0.002
Continuing training on VBD ^3^	2.22	0.511	−0.37–0.63	0.010
Knowledge of the sand flies	−0.65	0.517	0.33–0.80	0.003
Midwives (*n* = 21)				
Age (41–50 years)	−0.96	0.499	−0.12–2.30	0.035
Sex (Female)	2.43	0.486	0.43–5.10	0.014
Working structure	−0.17	0.415	0.52–1.35	0.473
Seniority in the health service (15–20 years)	0.83	0.435	−0.70–0.51	0.124
History of caring for people with cutaneous leishmaniasis	−0.10	0.461	0.55–1.46	0.676
Continuing training on VBD ^3^	−0.07	0.468	0.45–1.88	0.839
Knowledge of the sand flies	1.43	0.454	0.26–0.88	0.020
Health technicians (*n* = 8)				
Age (41–50 years)	−0.12	1.143	0.47–2.30	0.910
Sex (Male)	−2.81	0.950	−1.23–0.34	0.021
Work structure (Rural Health Centre)	−0.57	1.245	0.94–2.86	0.026
Seniority in the health service (15–20 years)	−2.21	0.410	−1.54–3.09	0.061
History of caring for people with cutaneous leishmaniasis	−0.07	1.031	0.45–1.88	0.839
Continuing training on VBD ^3^	0.69	1.016	−0.96–1.22	0.008
Knowledge of the sand flies	−0.77	0.470	0.21–0.99	0.049

^1^ OR: Odds ratio. ^2^ CI: Confidence interval. ^3^ VBD: Vector-borne diseases.

**Table 4:** The socio-demographic characteristics of the inhabitants, their levels of knowledge and their attitudes towards sand flies.

		*n*	%
Age (Year)	Age (Year) (Mean 38.84 ± 11.89 SD. Median 39 Years)		
	<20	17	6.0
	(20–30)	64	22.8
	(31–40)	70	24.9
	(41–50)	76	27.0
	>50	54	19.2
Sex	Female	191	68.0
	Male	90	32.0
Family situation	Single	46	16.4
	Married	210	74.7
	Widower	16	5.7
	Divorced	9	3.2
Education	Illiterate	88	31.3
	Primary	91	32.4
	Secondary	79	28.1
	Over	23	8.2
Activity/Occupation	Farming and husbandry	97	34.5
	Civil servant	19	6.8
	Daily/Employ	52	18.5
	Housewife	107	38.1
	Shopkeeper	6	2.1
Place of living	Rural	155	55.2
	Urban	126	44.8
District	Bouderbala	34	12.1
	Agourai	27	9.6
	Bitit	41	14.6
	Iqdar	34	12.1
	Laqsir	19	6.8
	ELHajeb	43	15.3
	SabaaAyoune	22	7.8
	AinTaoujdate	61	21.7
Monthly family income	No answer	27	9.6
	≤3000 DHS	147	52.3
	(3000–5000DHS)	69	24.6
	(5000–7000DHS)	28	10.0
	≥7000 DHS	10	3.6
Type of coverage	Without coverage	62	22.1
	CNOPS	24	8.5
	CNSS	45	16.0
	RAMED	150	53.4
Type of dwelling	Traditional house	135	48.0
	Modern house (cement)	121	43.1
	Apartment	23	8.2
	Villa	2	0.7
Have you ever been informed about insect-borne diseases?	Yes	182	64.8
	No	99	35.2
Overall knowledge of sand flies Adequate	Sufficient (score > 3)	86	30.6
	Insufficient (score ≤ 2)	195	69.4
Overall attitudes towards sand flies	Positive (≥11)	84	29.9
	Negative (<11)	197	70.1

**Table 5:** Factors associated with high knowledge and positive attitudes in the local community about sand flies.

Variable		Cœf.	OR ^1^	IC ^2^ à 95%	*p*-Value < 0.05
Sufficient knowledge	Age (41–50) years	1.10	1.17	0.190–1.980	0.011
	Sex (female)	0.57	1.07	0.013–1.037	0.021
	Level of education (high school)	−0.67	1.25	−1.29–0.09	0.030
	Living place (rural)	1.23	0.67	0.562–0.799	<0.001
	Already had (seen) lesions of cutaneous leishmaniasis	−0.21	0.80	−0.416–0.083	<0.001
	Ability to differentiate between mosquitoes and sand flies	−1.08	1.59	1.320–1.930	<0.001
Positive Attitudes	Education level (higher)	−1.45	1.04	−2.24–0.67	<0.001
	Be informed about diseases transmitted by insects	−0.25	0.77	0.65–0.91	<0.003
Sufficient knowledge and positive attitude		0.31	1.37	1.033–1.81	0.031

^1^ OR: Odds ratio. ^2^ CI: Confidence interval.
